# Predicting the risk of inappropriate depth of endotracheal intubation in pediatric patients using machine learning approaches

**DOI:** 10.1038/s41598-023-32122-5

**Published:** 2023-03-29

**Authors:** Jae-Geum Shim, Eun Kyung Lee, Eun Jung Oh, Eun-Ah Cho, Jiyeon Park, Jun-Ho Lee, Jin Hee Ahn

**Affiliations:** 1 Department of Anesthesiology and Pain Medicine, Kangbuk Samsung Hospital, Sungkyunkwan University School of Medicine, 29, Saemoonan-Ro, Jongro-Gu, Seoul, 03181 Republic of Korea; 2 Department of Anesthesiology and Pain Medicine, Chung-Ang University Hospital, Chung-Ang University School of Medicine, Seoul, Republic of Korea; 3 Department of Anesthesiology and Pain Medicine, Kwangmyeong Hospital, Chung-Ang University School of Medicine, Kwangmyeong, Republic of Korea

**Keywords:** Paediatric research, Paediatric research

## Abstract

Endotracheal tube (ET) misplacement is common in pediatric patients, which can lead to the serious complication. It would be helpful if there is an easy-to-use tool to predict the optimal ET depth considering in each patient’s characteristics. Therefore, we plan to develop a novel machine learning (ML) model to predict the appropriate ET depth in pediatric patients. This study retrospectively collected data from 1436 pediatric patients aged < 7 years who underwent chest x-ray examination in an intubated state. Patient data including age, sex, height weight, the internal diameter (ID) of the ET, and ET depth were collected from electronic medical records and chest x-ray. Among these, 1436 data were divided into training (70%, n = 1007) and testing (30%, n = 429) datasets. The training dataset was used to build the appropriate ET depth estimation model, while the test dataset was used to compare the model performance with the formula-based methods such as age-based method, height-based method and tube-ID method. The rate of inappropriate ET location was significantly lower in our ML model (17.9%) compared to formula-based methods (35.7%, 62.2%, and 46.6%). The relative risk [95% confidence interval, CI] of an inappropriate ET location compared to ML model in the age-based, height-based, and tube ID-based method were 1.99 [1.56–2.52], 3.47 [2.80–4.30], and 2.60 [2.07–3.26], respectively. In addition, compared to ML model, the relative risk of shallow intubation tended to be higher in the age-based method, whereas the risk of the deep or endobronchial intubation tended to be higher in the height-based and the tube ID-based method. The use of our ML model was able to predict optimal ET depth for pediatric patients only with basic patient information and reduce the risk of inappropriate ET placement. It will be helpful to clinicians unfamiliar with pediatric tracheal intubation to determine the appropriate ET depth.

## Introduction

Pediatric endotracheal intubation is mandatory in elective surgery and critically ill children^1,2^. However, endotracheal tube (ET) misplacement is common in pediatric patients with short tracheal lengths compared to adults; thus, it is challenging to determine the appropriate depth of ET^3–5^. Deep placement of the tip of the ET can irritate the carina, causing sympathetic stimulation. This may lead to tachycardia, hypertension, and bronchospasm^6^. Furthermore, endobronchial intubation may result in serious complications such as atelectasis, hypoxemia, barotrauma, and pneumothorax^7^. By contrast, shallow placement of the tip of the ET increases the risk of accidental extubation during manipulation of surgery or postural change^8^.

Several methods have been proposed to determine the appropriate ET placement. Methods for determining the appropriate depth of ET include age, height, ET internal diameter (ID)-based formulas, chest X-ray-based methods, and fiberoptic bronchoscopy^9,10^. Previous studies have shown that these methods have limitations in clinical practice due to the lack of accuracy of formula-based methods and the inconvenience of using the equipment.

### Application of machine learning techniques related to this study

With the development of machine learning approaches in recent years, a subfield of artificial intelligence which can automatically learn from big data and then artificially and independently make decisions upon the knowledge, now machine learning is widely applied to the medical field. Machine learning was applied to a broad range of problems in medicine such as medical diagnosis, medical treatment, medical education, and drug development including coronavirus research. For example, in radiological diagnosis, deep learning algorithm, a type of machine learning, was developed for brain tumor segmentation using MRI multi-modalities brain images^12^. Machine learning-based ultrasonographic image analysis, which was invented by Nguyen DT, showed he best performance for thyroid nodule classification^13^. In the field of medical treatment, machine learning technology also plays a crucial in the intensive care unit (ICU). For instance, the application of wireless sensor networks based on machine learning can be used to collect patient information, and reduce false alarms ensuring patient data security and patient safety, and lowering the power consumption and price^14^. Also, artificial intelligence could help scientists develop better medicines faster and improve clinical trial processes and cut down the research and development costs and time period^15^.

Recently, machine learning (ML) techniques have been applied to diverse areas in anesthesiology^16^. ML has shown relatively accurate performance in predicting and solving problems such as prediction of clinical outcomes and medical image classification^17,18^. ML-assisted individualized hemodynamic management in high-risk surgery can reduce the incidence and duration of intraoperative hypotension^19,20^. An endotracheal intubation training program using virtual reality (VR) has been developed in airway management by applying an ML technique through user feedback^21^. In addition, our previous study demonstrated that ML could predict the appropriate intubation depth more accurately than the conventional formula-based method in pediatric patients^22^. However, the sample size used in the study was very small. Thus, it was insufficient to verify the performance and clinical usefulness of the proposed ML model.

### Significance of the study

In this study, the ML approaches were employed for predicting endotracheal tube depth compared to formula-based methods based on age, height, and tube. To the best of our knowledge, this is the first study to predict the optimal endotracheal tube depth incorporating four basic patient information by using ML algorithms.

We have assumed that ML methods can reduce the risk of inappropriate ET positioning. Therefore, we have developed a new ML model for predicting the optimal endotracheal tube depth for actual intubation in pediatric patients. The primary objective of this study is to compare the inappropriate positioning incidence rates of the ML model and conventional formula-based methods. The secondary objective is to identify the relative risk of inappropriate locations in formula-based methods compared with the ML model.

## Methodology

This study retrospectively collected data from pediatric patients aged < 7 years who underwent chest X-ray examination in an intubated state at Samsung Medical Center from January 1, 2015 to Jan 30, 2022. The Institutional Board of Samsung Medical Center (Seoul, Korea; Chairperson Prof. Hojoong Kim, M.D.; Approval Number: SMC 2021-07-049; July 12, 2021) approved this study. The institutional review board waived the requirement for informed consent from patients owing to the retrospective design. The current study was conducted in accord with ethical guidelines of the Declaration of Helsinki of the world medial association. The exclusion criteria were tracheal or vertebral abnormalities, preoperative tracheostomies, and chest X-rays of insufficient quality.

### Data acquisition

Of 1573 pediatric patient records, 30 and 14 were excluded due to preoperative tracheostomies and low-quality chest X-rays, respectively, and 93 with missing data were also excluded. Finally, 1436 data records were used as the prediction model datasets. Patient data, including age, sex, height, weight, the internal diameter of the endotracheal tube (ET ID), and fixed ET depth, were collected from electronic medical records. The distance was measured using the caliper function of the picture archival and communication system (PACS, Centricity Enterprise Web V3.0, GE Healthcare, USA). For chest X-ray data, the distance from the carina to the upper margin of the first thoracic vertebral body (T1), distance from the carina to the lower margin of the third thoracic vertebral body (T3), and distance from the carina to the tip of the ET were measured using PACS. We assumed that the optimal location of the ET tip lies between the T1 upper margin and T3 lower margin^23,24^.

### Data preprocessing and data split

In resuscitation or general anesthesia, endotracheal tube depth is frequently estimated using the Advanced Pediatric Life Support (APLS) guidelines. Various methods have been described for accurate tube positioning including age, height, and ETT internal diameter (ID). Other study have shown that the length of trachea may vary according to gender^25^. Moreover, weight can be the independent variable for the length of the orotracheal tube in previous study^26^. Therefore, in this study, only baseline characteristics such as age, gender, height, and weight, which can be easily known before performing intubation, were selected as input values for the model.

**Figure 1:**
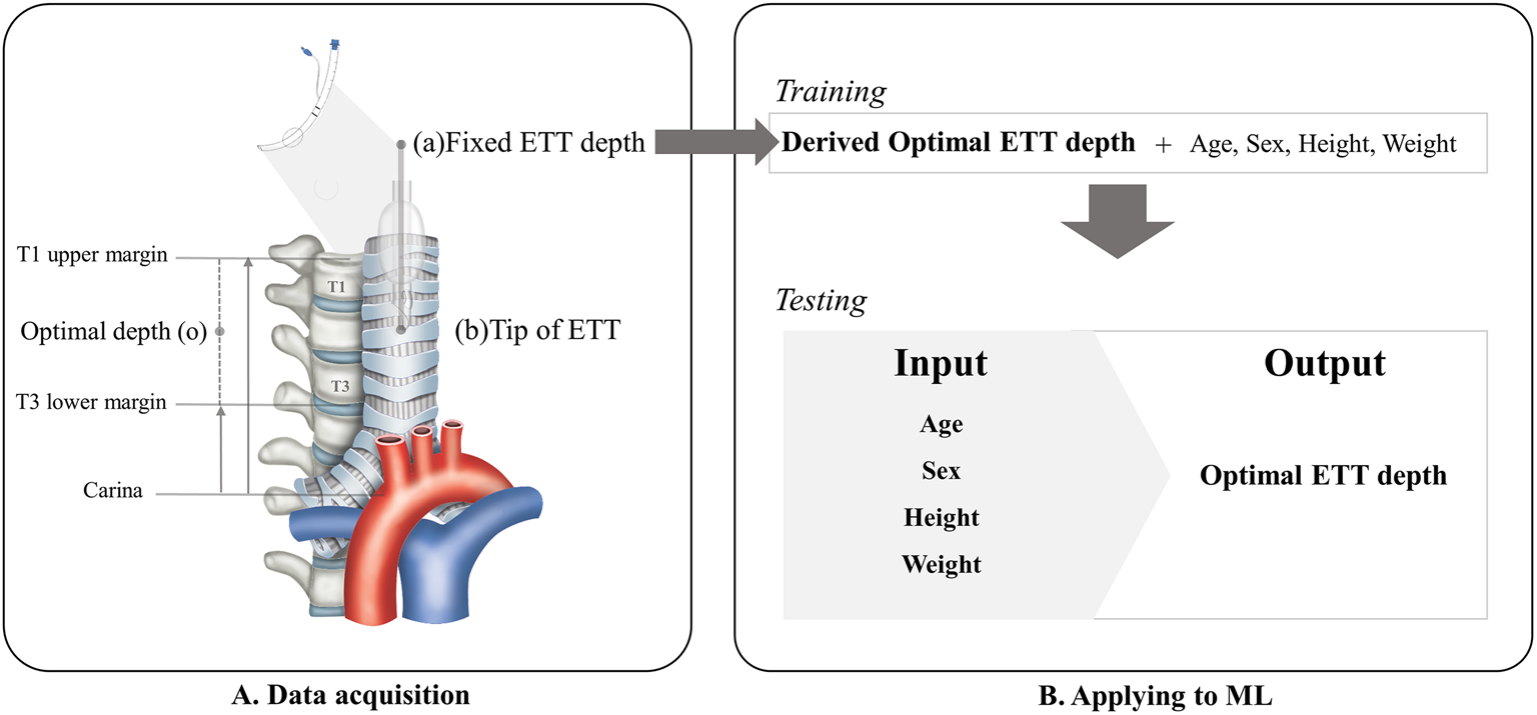
Process to estimate optimal depth of endotracheal tube using machine learning model. (**A**) Acquire the recorded value of (a) fixed ETT depth from the electronic medical record and measure the distance from the carina to the tracheal tube tip (b) on the chest X-ray. Then, assume that the median of the distance from the carina to the upper margin of T1 and the distance from the carina to the lower margin of T3 is the optimal tracheal tube tip position (C). Finally, the ETT optimal depth value is derived by moving (a) as much as the distance that (b) has moved to the position of (c). (**B**) Applying to machine learning model.

The dataset features included four variables: age, sex, height, and weight (Fig. 1). The target depth of ET was derived using the distance from the carina to the ET tube tip measured on chest radiography and the fixed depth value of ET on electronic medical records. When the tube tip was located at the center between the upper margin of T1 and the lower margin of T3, The fixed ET value was used as the target depth.

**Figure. 2:**
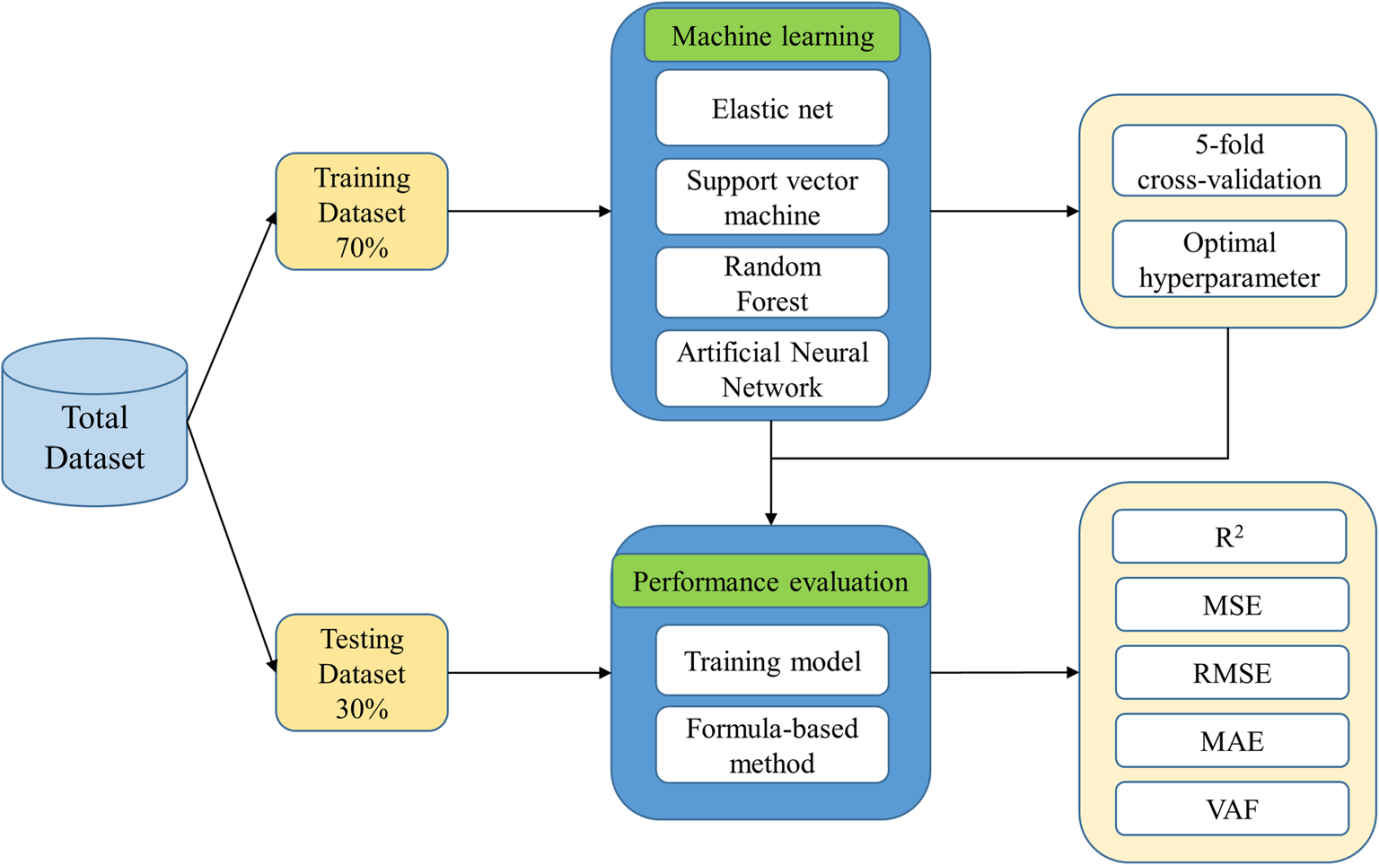
A flowchart of the work.

We separated 1436 records into training (1007/1436, 70%) and testing (429/1436, 30%) datasets (Fig. 2). We performed fivefold cross-validation to develop the ML prediction model. The training dataset was used to build the optimal ET depth estimation model, while the test dataset was used to compare the model performance with the formula-based methods. We standardized the training set to maintain the parameters (mean and standard deviation values, respectively) for each feature. These parameters are applied to transform the test set.

### Prediction model development

#### Elastic net

We used an elastic net to develop the ML model for predicting the optimal depth of endotracheal intubation^27,28^. Regression is widely used to form a model to predict numeric values. Simple linear regression analysis is a technique that assumes a linear relationship between inputs and the target variable. Regularization was added to the loss function of linear regression to enhance the prediction accuracy and interpretability of the resulting predictive model with smaller coefficient values. Elastic net is a regularized regression method that linearly combines two popular penalties, specifically ridge and lasso functions to prevent overfitting the model.^29^. This is also known as a shrinkage and selection method which contains both the L_1_ and L_2_ penalties of the Lasso and Ridge regression. Imposing this penalty means that the coefficient values are reduced towards zero allowing the less contributive variables to have a coefficient close to zero (like ridge regression) or equal zero (like lasso regression). We implemented the models using R, version 4.1.2 (The R Foundation)^30^.

The elastic net allows tuning of the penalty term of the Eq. (1) requiring the selection of a tuning parameter (α, lambda).

1 $$\underset{\left({\beta }_{0},{\varvec{\beta}}\right)}{\mathrm{min}}{\left(y- {\beta }_{0}-{{\varvec{X}}}^{T}{\varvec{\beta}}\right)}^{2}+ \lambda \left(\frac{1}{2}\left(1- \alpha \right){{\varvec{\beta}}}^{2}+ \alpha \left|{\varvec{\beta}}\right|\right)$$

The parameter α determines the type of shrinkage, with Lasso (α = 1 in the equation above) or Ridge (α = 0) regression. A value of α is set between 0 and 1. The amount of shrinkage can be fine-tuned using the penalty parameter λ called lambda (λ).

### Prediction of ET depth

#### Machine learning model

We provided the inputs, including age, sex, height, weight, and the target depth of ET, as the training data in the elastic net algorithm. Therefore, if new patient data (age, sex, height, and weight) were entered into the built model, the ET depth was derived such that the ET was located at the optimal depth.

#### Formula-based methods

For the formula based on age, the insertion depth was estimated to be 9 cm for those aged 0–6 months, 10 cm for those aged 6–12 months, and 11 cm for those under 2 years^31^. In children older than 2 years, the tube depth was estimated using the pediatric advanced cardiac life support formula (tube depth [cm] = 12 + age [years]/2)^32,33^. For the height-based formula, we used the Morgan and Steward formula (tube depth [cm] = height [cm]/10 + 5)^34^. For the tube ID-based formula, the insertion depth was estimated using the formula: 3 x (tube ID)^35^.

### Performance evaluation

Appropriate predictions of ET depth were defined as ET placed between the T1 upper margin and the T3 lower margin; any other location was an inappropriate prediction. Inappropriate predictions were divided into shallow, deep, and bronchial intubations. Deep intubation was defined as when the tip of the ET was located below the lower border of T3. Bronchial intubation was defined when the tip of the ET was located below the carina. Conversely, if the tip of the ET was located above the T1 upper border, it was defined as shallow intubation.

### Performance indices

The Performance of each model was evaluated using five well-known indices i.e., coefficient of correlation (R^2^), mean square error (MSE), root mean square error (RMSE), mean absolute error (MAE), and variance account for (VAF)^36,37^. The following Eqs. (2)–(6) represent the formula that was used for calculating the mentioned performance indices for testing data^38,39^.

2 $${R}^{2}= 1- \frac{{\sum }_{i=1}^{n}{\left(y-y{^{\prime}}\right)}^{2}}{{\sum }_{i=1}^{n}{\left(y-\overline{y }\right)}^{2}}$$

3 $$\mathrm{SE}= \frac{1}{n}\sum_{i=1}^{n}{\left(y- \overline{y }\right)}^{2}$$

4 $$\mathrm{RMSE}=\sqrt{\frac{1}{n}\sum_{i=1}^{n}{\left(y- \overline{y }\right)}^{2}}$$

5 $$\mathrm{MAE}= \frac{1}{n}\sum_{i=1}^{n}\left|y-\overline{y }\right|$$

6 $$\mathrm{VAF}= \left[1- \frac{var\left(y-y{^{\prime}}\right)}{var\left(y\right)}\right]\times 100$$

where y depicts the measured values, $\overline{y }$ and y’ indicate mean and predicted of the y, respectively, n is the total number of data.

### Statistical analysis

Summary of descriptive statistics includes frequencies (%) and medians [interquartile ranges. Student’s t-test, Wilcoxon rank-sum test, Pearson’s χ^2^ test, and Fisher’s exact test analyzed the differences between the training and test sets. Relative risks and 95% confidence intervals (CIs) were calculated depending on the scenario. The relative risk and 95% CIs, excluding 1, were considered statistically significant. Differences and 95% CIs, excluding 0, were considered statistically significant. All statistical analyses were performed using MedCalc® Statistical Software version 20.014 (MedCalc Software Ltd, Ostend, Belgium; https://www.medcalc.org; 2021) and R, version 4.1.2 (The R Foundation). We used 2-tailed tests in all analyses, with *P* values < 0.05 considered statistically significant.

## Result and discussion

In this retrospective study, we evaluated 1573 pediatric patients for eligibility, from which 134 records were excluded, as discussed in the above sections. Therefore, 1436 patients were separated into training (70%, n = 1007) and test (30%, n = 429) sets. Patient characteristics are shown in Table 1. The patient characteristics were not statistically different between the training and test datasets.

**Table 1:** Patient demographic data and variable features.

Variables	Total, *n* = 1436(100%)	Training set, *n* = 1007(70%)	Test set, *n* = 429(30%)	*p*-Value
Age				0.57
< 6 months	845 (58.8)	599 (59.5)	246 (57.3)	
6 months—1 year	154 (10.7)	106 (10.5)	48 (11.2)	
1–2 years	254 (17.8)	166 (16.5)	88 (20.5)	
2–7 years	183 (12.7)	136 (13.5)	47 (11.0)	
Height (cm)	62 [52, 80]	62 [52, 80]	62 [53, 79]	0.82
Weight (kg)	6.0 [3.8, 10.1]	5.8 [3.7, 10.2]	6.1 [4.0, 10.0]	0.07
Sex (female)	705 (49.1)	494 (49.1)	211 (49.2)	1.0
Tube size (ID in mm, %)				0.13
2.5	5 (0.4)	3 (0.3)	2 (0.5)	
3.0	293 (20.4)	209 (20.8)	84 (19.6)	
3.5	539 (37.5)	392 (38.9)	147 (34.3)	
4.0	328 (22.8)	207 (20.6)	121 (28.2)	
4.5	183 (12.7)	132 (13.1)	51 (11.9)	
5.0	54 (3.8)	40 (3.9)	14 (3.2)	
5.5	28 (2.0)	19 (1.9)	9 (2.1)	
6.0	6 (0.4)	5 (0.5)	1 (0.2)	
Distance from carina to T1 (cm)	3.1 [2.6, 4.2]	3.1 [2.6, 4.1]	3.2 [2.6, 4.2]	0.76
Distance from carina to T3 (cm)	0.8 [0.5, 1.2]	0.8 [0.5, 1.2]	0.8 [0.5, 1.3]	0.45
Distance from carina to tracheal tube tip (cm)	1.2 [0.8, 1.7]	1.2 [0.8, 1.6]	1.2 [0.8, 1.7]	0.42

The postoperative chest X-ray measured the distance from the carina to the T1 upper border, T3 lower border, and the tracheal tube tip.

The data are presented as median [interquartile range] or number (%).

ID: internal diameter.

We evaluated the prediction performance of the elastic net model with the isolated testing data set (n = 429). To compare the prediction performance of our proposed model with those of other ML models, we separately trained the following models: support vector machine, random forest, and artificial neural network. We evaluated the prediction performance of these ML models as well as formula-based methods.

**Figure 3:**
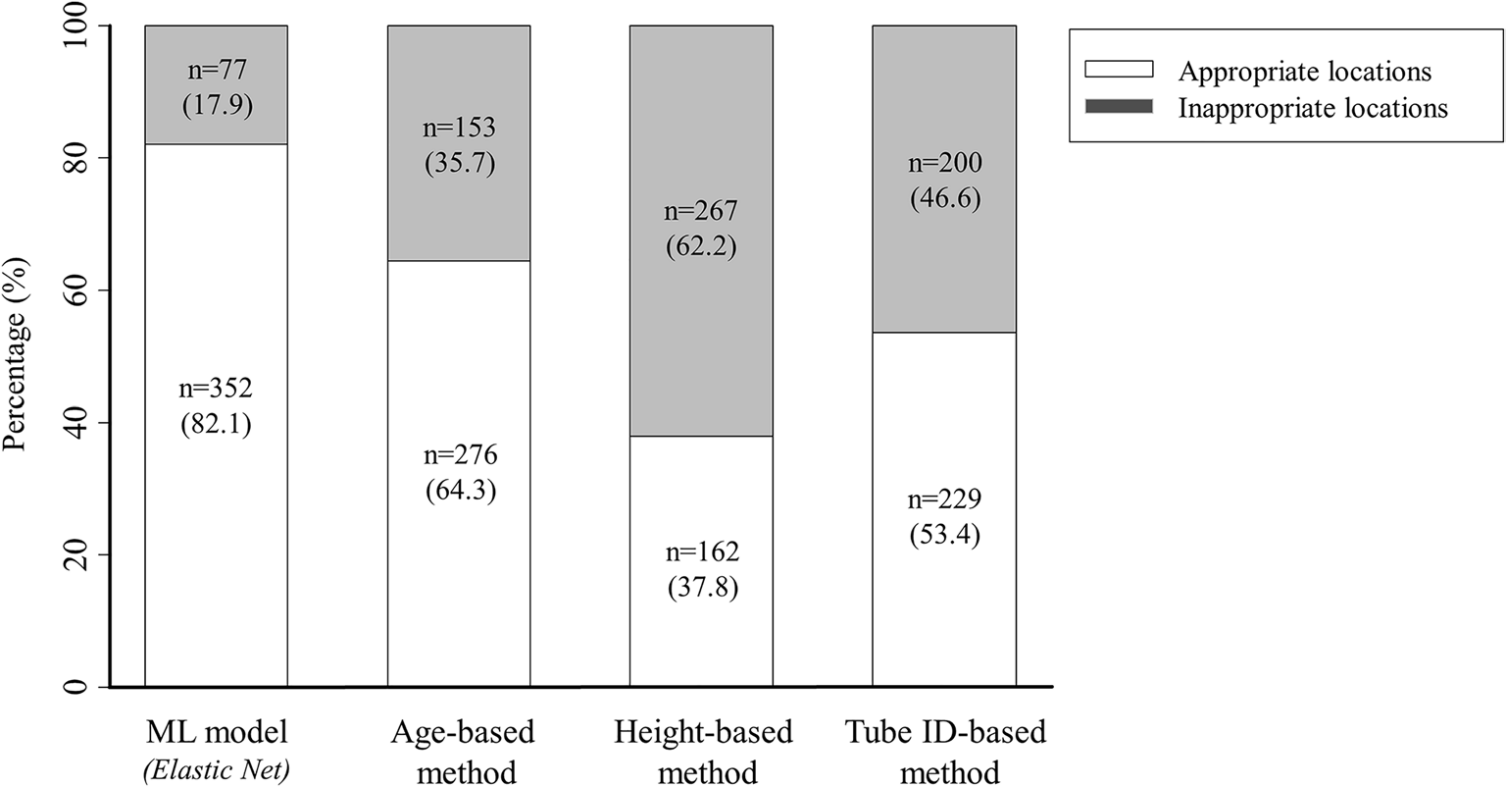
Percentages of appropriate and inappropriate locations of the ET tip in the machine learning model and formula-based methods.

The values of the performance indices of the models are presented in Table 2. The results of the evaluation showed that the elastic net model predicts the optimal depth of endotracheal intubation with lower MSE, RMSE, and MAE, and with a high R^2^ and VAF in test data. The frequency and percentage of the test datasets predicting the appropriate and inappropriate depth prediction for each method are presented in Table 3 and Fig. 3. The frequency (rate) of inappropriate locations in the machine learning model was 77 (17.9%). The frequency (rate) of inappropriate places was 153 (35.7%), 267 (62.2%), and 200 (46.6%) for the age-based, height-based, and tube ID-based method, respectively. In addition, the relative risk [95% confidence interval, CI] ET location in an inappropriate position in the age-based method, height-based method, and tube ID-based method were 1.99 [1.56–2.52], 3.47 [2.80–4.30], and 2.60 [2.07–3.26], respectively compared to ML models (Table 4). The formula-based method's relative risk of being in an inappropriate position was significantly higher.

**Table 2:** Performance indices of the proposed algorithms in this study.

	R^2^	MSE	RMSE	MAE	VAF
Machine learning method					
Elastic Net	66.355	1.000	1.000	0.743	66.338
Support vector machine	52.081	1.001	1.000	0.748	66.495
Random forest	46.085	1.122	1.059	0.786	62.078
Artificial neural network	39.543	1.175	1.084	0.837	63.699
Formula-based method					
Age-based method	46.598	1.979	1.407	1.051	34.847
Height-based method	54.109	3.891	1.973	1.624	41.953
Tube ID-based method	55.165	2.361	1.536	1.198	50.309

Perfect R^2^ = 100%; Perfect MSE = 0; Perfect RMSE = 0; Perfect MAE = 0; Perfect VAF = 100%

The values of the performance indices of the models are presented in Table 1. The results of the evaluation showed that the elastic net model predicts the optimal depth of endotracheal intubation with lower MSE, RMSE, and MAE, and with a high R^2^ and VAF in test data.

**Table 3:** Classification of inappropriate ET depth.

	Total inappropriate depth, n (%)	Shallow intubation, n (%)	Deep intubation, n (%)	Endobronchial intubation, n (%)
Machine learning model				
Elastic net	77 (17.9)	29 (6.8)	44 (10.3)	4 (0.9)
Support vector machine	80 (18.6)	29 (6.8)	46 (10.7)	5 (1.2)
Random forest	96 (22.4)	37 (8.6)	52 (12.1)	7 (1.6)
Artificial neural network	90 (21.0)	67 (15.6)	21 (4.9)	2 (0.5)
Formula-based method				
Age-based method	153(35.7)	94 (21.9)	40 (9.3)	9 (2.1)
Height-based method	267(62.2)	4 (0.9)	209 (48.7)	53 (12.4)
Tube ID-based method, n (%)	200(46.6)	14 (3.3)	140 (32.6)	46 (10.7)

Data are presented as number(percentage).

**Table 4:** Relative risk of inappropriate predictions in formula-based methods compared to the ML model.

	Inappropriate depth	*P*-value	Shallow intubation	*P*-value	Deep intubation	*P*-value	Endobronchial intubation	*P*-value
Age-based method	1.99 [1.56–2.52]	< 0.0001	3.34 [2.26–4.93]	< 0.0001	1.14 [0.76–1.70]	0.5249	2.81 [0.87–9.03]	0.0828
Height-based method	3.47 [2.80–4.30]	< 0.0001	0.32 [0.11–0.89]	0.0285	5.07 [3.78–6.79]	< 0.0001	21.94 [8.05–59.76]	< 0.0001
Tube ID-based method	2.60 [2.07–3.26]	< 0.0001	0.76 [0.41–1.14]	0.3765	3.41 [2.51–4.64]	< 0.0001	14.89 [5.42–40.86]	< 0.0001

Data are presented as risk ratio [95% confidence interval].

Inappropriate ET depths were categorized into shallow, deep, and endobronchial intubations, as shown in Table 3. In the ML model, shallow, deep, and endobronchial intubation rates were 6.8%, 10.3%, and 0.9%, respectively. Among formula-based methods, shallow placement was frequently observed in the age-based method, and deep and endobronchial placement was commonly observed in the height-based and tube ID-based methods. The relative risk [95% CI] of ET location in the shallow was higher as 3.34 [2.26–4.93] in the age-based method compared to the ML model. By contrast, the relative risk of ET location in the deep and endobronchial placement was significantly higher in the height-based method and the tube ID-based method (Table 4).

**Figure 4:**
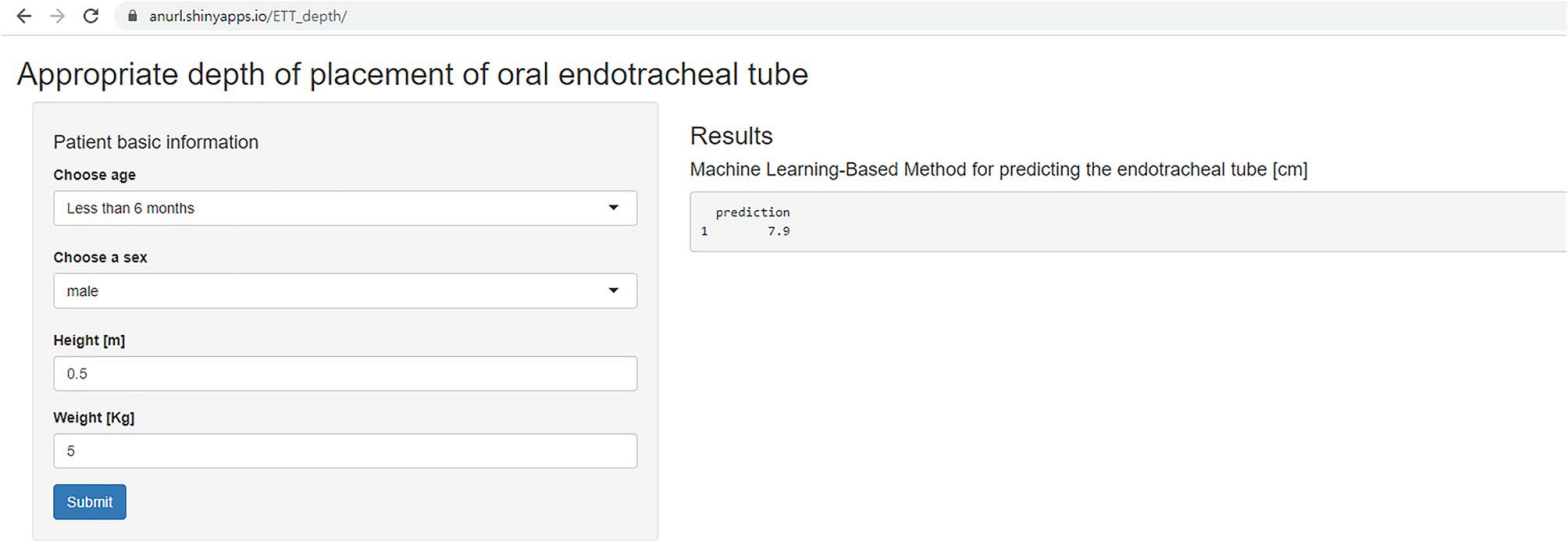
Screenshot of the web application.

Additionally, we have developed a web application to predict pediatric patients’ appropriate depth based on the proposed ML model (Fig. 4., web address: https://anurl.shinyapps.io/ETT_depth).

Conventional formula-based methods calculated the insertion depth of the ET based on only one specific variable. Morgan's height formula assumes that the length of the trachea is proportional to the height of the pediatric patient. That height is considered the most critical factor in determining ET depth^34^. Although the trachea length and height were correlated, the rate at which the appropriate ET depth was observed was only 43%^23,40^. In addition, in the weight-based method proposed by Gill et al., the ET tube was located in inappropriate positions in 56% of cases, in deep positions in 52% of patients^41^. The prediction of ET depth using a single biological variable cannot be applied to actual clinical practice due to the low accuracy. Therefore, it is essential to develop a new model for estimating the insertion depth of ET considering the various characteristics of pediatric patients.

The proposed ML model can derive the ET depth by considering four variables: age, gender, height, and weight. As the input values consist of fundamental patient information, it is easy to obtain the required information. In addition, the input value of the optimal ET depth obtained from the chest X-ray, regarded as the gold standard for determining the ET depth, can improve the accuracy of the results. The prediction accuracy was in the range of 38–64% with previous a single variable but increased to 82% with four variables. Furthermore, the relative risk of ET located at an inappropriate depth (shallow, deep, and endobronchial) was significantly lower in the ML model than in formula-based methods.

An ML model is helpful for anesthesiologists who are unfamiliar with pediatric airway management. Artificial intelligence (AI) uses advanced mathematical models to find connections into complex data determined by human intelligence alone. An ML-based intubation-assisted device could identify the creation of label vocal cords and tracheal rings in the airway tract on a real-time video screen, and the sensitivity and specificity were up to 89% and 98%^42^. In this study, we have also developed an ML model as a web application so that anyone can easily access the prediction model and predict the appropriate ET depth. A potential future application of AI can be recognizing the airway structure of each patient and guiding the proper ET depth in pediatric airway management^43^.

## Conclusion

The appropriate insertion depth of an endotracheal tube (ET) should be determined for pediatric patients because both deep and shallow intubation may result in serious complications related to endotracheal tube misplacement. It would be helpful if there is an easy-to-use tool to predict the optimal ET depth considering in each patient's characteristics. Therefore, we developed and validated ML models to predict the appropriate ET depth in pediatric patients. The proposed ML model accurately predicted the appropriate depth of the ET with adequate accuracy (83%) compared to formula-based methods in pediatric patients below seven years. In addition, our study results demonstrated that the proposed model can reduce the risk of shallow or deep intubation and endobronchial intubation. Applying an ML model can be safe and helpful in determining the appropriate ET depth for pediatric patients.

There are some limitations to this study. First, there may be a difference between the time points of ET fixation and chest radiography. The ET position may not have been the initial depth because the tube position can change after fixation. According to the ICU processes of our institution, during the postoperative period, a chest X-ray examination was performed immediately after confirming tube fixation, and we could use the data that minimized the time difference. Second, it has not been applied to actual ET intubation in pediatric patients. Compared to previous studies, this study used a large amount of data and showed satisfactory performance; however, an actual clinical application process is required for generalization.

In future work, prospective studies may allow comparison of our ML approach to the alternative strategy for clinical assessment. Though the introduction of our proposed elastic net model showed reasonable accuracy, our study used only single center data to develop prediction model. Thus, external validation to verify ML performance is needed. We plan to update and test our model using data from other external institutions in further studies.

A “Key Points” summary is provided, which describes outcome and findings from this study.

- The elastic net model showed its ability to predict the optimal depth of endotracheal intubation with lower MSE, RMSE, and MAE, and with a higher R2 and VAF compared to other models in test data.
- The frequency rate of inappropriate locations in the elastic net model was 17.9% in test data. In the elastic net model, shallow, deep, and endobronchial intubation rates were 6.8%, 10.3%, and 0.9%, respectively.
- This will be helpful in decision-making regarding ET depth to clinicians unfamiliar with tracheal intubation in pediatric patients. However, an actual clinical application process is required for generalization.

## Data Availability

The datasets used and analyzed during the current study are available from the corresponding author on reasonable request.
